# A Beam Hardening Artifact Correction Method for CT Images Based on VGG Feature Extraction Networks

**DOI:** 10.3390/s25072088

**Published:** 2025-03-26

**Authors:** Hong Zhang, Zhaoguang Ma, Da Kang, Min Yang

**Affiliations:** 1 School of Mechanical Engineering and Automation, Beihang University, Beijing 100191, China; zh31sbuaa@163.com; 2 Beijing Power Machinery Research Institute, Beijing 100074, China; hmguang_24@163.com (Z.M.); kang_da@126.com (D.K.)

**Keywords:** industrial CT, beam hardening correction, aerospace blades, deep convolutional neural network

## Abstract

In X-ray industrial computed tomography (ICT) imaging, beam hardening artifacts significantly degrade the quality of reconstructed images, leading to cupping effects, ring artifacts, and reduced contrast resolution. These issues are particularly severe in high-density and irregularly shaped aerospace components, where accurate defect detection is critical. To mitigate beam hardening artifacts, this paper proposes a correction method based on the VGG16 feature extraction network. Continuous convolutional layers automatically extract relevant features of beam hardening artifacts, establish a nonlinear mapping between artifact-affected and artifact-free images, and progressively enhance the model’s ability to understand and represent complex image features through stacked layers. Then, a dataset of ICT images with beam hardening artifacts is constructed, and VGG16 is employed to extract deep features from both artifact-affected and reference images. By incorporating perceptual loss into a convolutional neural network and optimizing through iterative training, the proposed method effectively suppresses cupping artifacts and reduces edge blurring. Experimental results demonstrated that the method significantly enhanced image contrast, reduced image noise, and restored structural details, thereby improving the reliability of ICT imaging for aerospace applications.

## 1. Introduction

**Figure 1:**
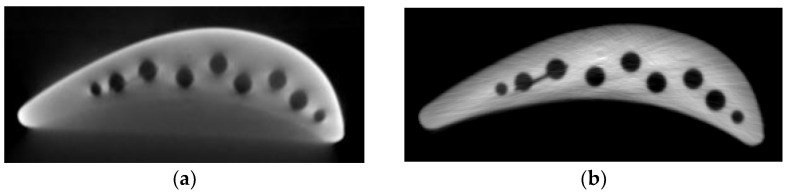
Comparison of reconstructed CT images of turbine blades before and after correction. (**a**) Image with beam hardening artifacts; (**b**) corrected image.

The X-rays produced by CT tubes are not monochromatic but have a certain spectral width. When high-attenuation materials such as metal are present in the CT scan field, low-energy photons in the X-ray spectrum are absorbed by the metal, causing some photons to fail to reach the detector, while higher-energy X-rays more easily penetrate the material. As the average energy of the X-rays increases during propagation, the rays become more penetrating or “harder.” This leads to the beam hardening effect under the combined influence of the beam spectrum’s polychromaticity, attenuation, and energy dependence [1]. Beam hardening artifacts in CT reconstructed images appear as areas that are dark in the center with bright edges, where the gray-level curve of the middle layer in the CT image exhibits a “cupping” shape, as shown in Figure 1. These artifacts severely affect the interpretability of CT images.

The material of the aerospace structural components studied in this paper is titanium alloy. In engineering applications, the polychromatic X-rays passing through this material exhibit a particularly pronounced hardening effect, resulting in distinct “hardening artifacts” in the reconstructed images. Current methods for beam hardening artifact correction primarily include four categories: filter-based correction [2], polynomial correction [3], iterative correction [4], and dual-energy correction [5].

By using filters to reduce the intensity of soft X-ray beams, artifact interference caused by the hardening effect can be effectively suppressed [6]. For example, Tan et al. conducted experiments with copper filters, which were able to mitigate beam hardening artifacts to a certain extent [7]. Zeng et al. studied a polynomial fitting beam hardening correction method based on experimental spectra and Monte Carlo simulations, effectively reducing the impact of the hardening effect on imaging quality [8].

The polynomial correction method is based on the characteristics of the beam hardening effect, which causes a nonlinear relationship between the projection values and the transmission path length. To address this issue, the method performs inverse correction through linearization, restoring the linear relationship between the projection values and the X-ray transmission length. Ultimately, this correction process effectively mitigates the beam hardening effect, ensuring more accurate transmission data and improving imaging precision. Kyriakou et al. utilized threshold segmentation to extract regions of interest from images and calculated the correspondence between the projection values and the transmission path length, thereby determining the parameters of the polynomial [9].

The iterative correction method integrates the beam hardening correction process into the reconstruction model, allowing for dynamic adjustment of the ray data. This method is typically divided into two categories: algebraic algorithms and statistical algorithms. De Man et al. utilized the characteristic of photon detection following a Poisson distribution and applied a statistical iterative method to correct CT images, resulting in more accurate reconstruction outcomes [10]. Lin et al. assumed that the human body is composed of several basic materials and proposed a beam hardening artifact correction algorithm that incorporates the simultaneous algebraic reconstruction technique (SART) [11].

The dual-energy correction method is based on the interaction between X-rays and materials, as well as the compositional characteristics of the base material. It decomposes the attenuation coefficient into two physical quantities that are energy-dependent [12,13]. By using projection data obtained from high-energy and low-energy spectra, the decomposition coefficients are calculated, enabling the estimation of the material’s attenuation coefficient μ at any energy level. Zhang et al. developed a method based on the H-L (high–low energy transmission attenuation value) curve, using a lookup table to achieve the decomposition of dual-energy projections into basis function projections and then employing the traditional FBP reconstruction algorithm for image reconstruction [14]. Alvarez et al. proposed a new dual-energy CT projection decomposition method that improves the accuracy of projection decomposition based on the projection matching principle [15]. With advancements in X-ray sources, detector technologies, and reconstruction theories, dual-energy CT has gradually become a research hotspot in the field of X-ray inspection.

Deep learning technologies have shown outstanding performance in image feature extraction and data fusion, with an increasing number of studies combining convolutional neural networks (CNNs) with medical image processing [16,17,18,19,20,21,22,23], achieving promising results. In 2016, Zhang et al. [24] proposed a method for correcting streak artifacts under undersampling by using CNNs to extract artifact features and suppressing them through nonlinear filtering, thereby improving the quality of images reconstructed using the FBP algorithm under such limited conditions. In 2017, Chen et al. [25] introduced two different network structures based on deep learning technology, which, without the need for original projection data, processed the reconstructed images to suppress noise in low-dose CT. In 2018, Chen et al. [26] incorporated deep learning into compressed sensing imaging algorithms, using networks to learn regularization parameters in iterative reconstruction, improving image quality while reducing computational complexity by several orders of magnitude. In 2018, Zhang et al. [27] proposed an open neural network architecture that utilized convolutional neural networks as a medium for information fusion to correct metal artifacts in CT images. These research advances demonstrate the immense potential of deep learning in medical image processing, particularly in feature extraction and information fusion. Kailash [28] proposed an artificial-intelligence-based solution, utilizing a cascaded deep neural network (CDNN) architecture constructed with convolutional layers, ReLU, and batch normalization layers, combined with skip connections to enhance feature learning. The CDNN leverages Fourier-transform-based reconstructed images as a prior, effectively reducing beam hardening artifacts in limited-angle CT. This method is primarily applied to image artifacts caused by limited-angle reconstruction.

Although beam hardening artifacts are a traditional problem in the field of CT and have been effectively addressed in certain areas, such as medical CT imaging, the beam hardening artifacts in CT images of high-density alloys have not been well resolved, and the relevant literature is still limited. Therefore, it is necessary to explore the mechanisms behind the formation of these complex artifacts and develop correction methods suitable for high-density metallic materials in aerospace applications. In Section 2, we introduce the method employed in this paper. Section 3 covers data acquisition and experimental parameter settings. Section 4 demonstrates the beam hardening artifact correction effect of the proposed algorithm through experiments with simulated and actual data. Section 5 presents the conclusion of the paper.

## 2. Beam Hardening Artifact Correction Method Based on Feature Extraction

This paper focuses on the correction of beam hardening artifact images. Currently, there are two main methods for correcting beam hardening in single-material samples: dual-energy CT and linear correction. The dual-energy CT correction method utilizes energy spectrum information and plays a significant role in correcting beam hardening artifacts in multimaterial samples. However, the X-ray energy spectrum is often estimated through indirect measurement data, which means fluctuations in projection data noise can inevitably affect the final reconstruction results, leading to a reduced signal-to-noise ratio in the reconstructed images. Additionally, because of its low detection efficiency, dual-energy CT is limited in industrial applications. The linear correction method typically requires phantoms made from the same material as the sample being inspected, offering limited flexibility and needing further improvement in its correction effectiveness. It is also prone to amplifying noise signals in the image.

The image domain postprocessing method leverages deep neural networks for end-to-end learning and processing of images, enabling more efficient and accurate image processing tasks. By using deep convolutional networks to automatically extract relevant features from beam hardening artifact images, a mapping relationship with artifact-free ground truth images is established, resulting in high-quality images without artifacts. This paper proposes a beam hardening artifact correction algorithm based on the VGG feature extraction network. The core idea of the algorithm is to build a dataset by simulating beam hardening tomography images of numerous material samples and learning multidimensional artifact features from them to train the network model. To enhance the model’s generalization ability, various energy conditions and sample material types are employed during the simulation of beam hardening artifact images. Furthermore, this paper provides a detailed description of the network architecture and the design of the loss function, and the suppression effect of the network on beam hardening artifacts was validated through tests on simulated and real images.

### 2.1. The Overall Structure of the Algorithm

**Figure 2:**
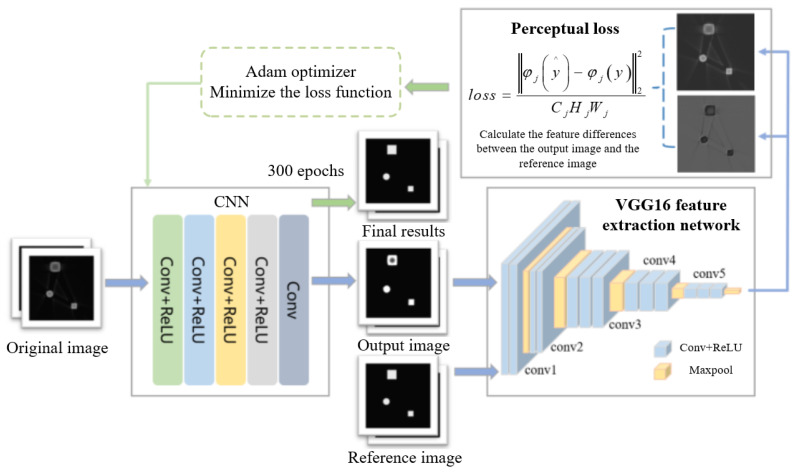
The flow chart of beam hardening artifact correction method based on feature extraction network.

The processing flow of the beam hardening artifact correction algorithm is shown in Figure 2. The core of the algorithm consists of a convolutional neural network and a VGG16 feature extraction network, which are connected via a loss function for data transmission. First, the original image containing artifacts is input into the convolutional neural network for processing. After passing through multiple network layers, a corrected tomography image is obtained. To more effectively remove artifacts and accurately restore the object’s edge contours, the VGG16 feature extraction network is introduced before calculating the loss value. This network fully extracts the features of the reference image and the output image. The perceptual loss is then calculated based on these feature maps and fed back into the convolutional neural network. Through multiple iterations and updates, the network thoroughly learns the image features, resulting in a beam hardening artifact-corrected image that maximizes the preservation of the object’s edge structure in the original image.

The beam hardening artifact correction network consists of an input layer, five convolutional layers, and an output layer. The network takes a 512 × 512 size simulated beam hardening image as the initial input. The continuous convolutional layers serve several purposes: (1) automatically extracting relevant features of the beam hardening artifacts using deep neural networks; (2) associating the artifact features with those of artifact-free images to establish a nonlinear mapping relationship; (3) stacking convolutional layers progressively, which helps the network extract increasingly complex and abstract features, thereby enhancing the model’s ability to understand and represent the image. This layer-by-layer processing allows the network to build a comprehensive image feature representation, starting from low-level features such as edges and textures and advancing to high-level features such as the shape and structure of objects.

To further accelerate the network’s convergence speed and enhance its nonlinear expression capabilities, this paper introduces the ReLU activation function after the first four convolutional layers. The ReLU activation function removes negative values while introducing nonlinearity to the model, helping improve its ability to fit complex patterns. The relationships between the image features extracted by the convolutional layers can be expressed as follows:

(1) $$P_{n}\left(x\right)=RELU\left(W_{n}*P_{n-1}\left(x\right)+b_{n}\right),n=1,2,\ldots N-1$$

where $P_{n}\left(x\right)$ and $P_{n-1}\left(x\right)$ represent the output of the current layer and the output of the previous layer, respectively; x represents the input image; n denotes the layer number within the network; and $W_{n}$ and $b_{n}$ are the weight and bias of the n−th layer, which are continuously updated during the network training process, thereby achieving the goal of minimizing the loss function.

### 2.2. The VGG-Net Feature Extraction Network

To ensure that the generated corrected image closely resembles the reference image, it is necessary to extract the features of both the generated image and the reference image, incorporating these features into the calculation of the objective function. Through the feedback mechanism of the loss function and optimization algorithm, the generated image is ensured to be semantically similar to the reference image. Several mature feature extraction networks are available for use, such as AlexNet [29] and ResNet [30]. These networks typically use larger convolution kernels and shallower network layers to avoid the issue of excessive parameters. In subsequent deep learning network research, Goodfellow et al. proposed that the greater the depth of the network’s hidden layers, the higher the fitting accuracy. The network was proposed by the Visual Geometry Group (VGG) from the University of Oxford and is commonly referred to as VGG. The VGG network performed exceptionally well in the 2014 ImageNet image recognition challenge. The structure of the VGG network is both simple and classic, consisting primarily of a series of convolutional layers and pooling layers, followed by several fully connected layers. The convolutional layers in the VGG network use smaller 3 × 3 convolution kernels and are stacked, which helps retain more local information and improves the network’s nonlinear representation capability. Between the convolutional layers, the VGG network employs max-pooling layers to reduce the dimensionality of feature maps while preserving important features. After the convolutional and pooling layers, fully connected layers are used for classification. A key feature of the VGG network is its relatively large depth, with numerous convolutional layers and parameters, but its structure remains simple, with highly regular connections between layers. Depending on the network depth, it is divided into two variants: VGG16 and VGG19.

In this paper, VGG16 was selected as the backbone structure of the image feature extraction network. The VGG16 network consists of 13 convolutional layers, 5 max-pooling layers, and 3 fully connected layers. The primary function of the max-pooling layers is to reduce the dimensionality of matrices. However, in this algorithm, the fully connected layers are removed, because the task of suppressing image artifacts does not require converting image features into specific numerical values. Instead, the feature maps are directly input into the perceptual loss function for subsequent structural difference value calculations. The feature extraction network structure used in this paper is shown in Table 1.

The VGG16 network structure is composed of five modules, each containing 3 × 3 convolutional kernels and max-pooling operations with a 2 × 2 pixel window. When the simulated single-material metal images are input into this network, multiple feature maps at different depths of convolutional layers can be obtained.

**Figure 3:**
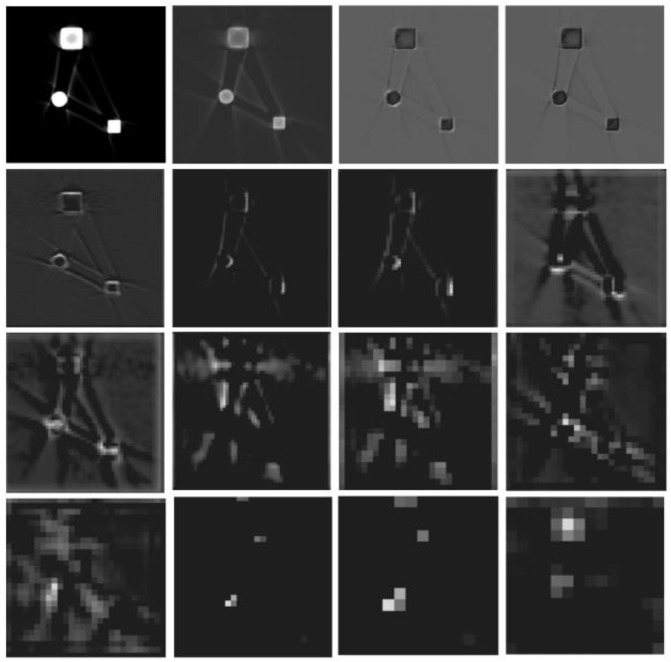
Simulation image and feature extraction map of beam hardening artifacts.

Figure 3 shows an original artifact image and the corresponding feature maps. The top-left corner displays the original image, followed by feature maps from different depths of the network arranged sequentially from left to right and top to bottom. Observing this figure reveals that as the network depth increases, the extracted image features become progressively more abstract. In the final layers of the network, the feature map dimensions are significantly reduced because of pooling. For better visualization, all feature maps at different layers are resampled to a uniform size in this paper.

### 2.3. Loss Function

In deep-learning-based image reconstruction tasks, the mean squared error (MSE) is commonly used as the loss function for network training. However, MSE is highly sensitive to noise and outliers, which can lead to unstable reconstruction results. Additionally, directly using the MSE loss function to compare generated and reference images often results in overly smooth images, with degraded quality in terms of fine details, e.g., blurring.

This paper adopts the perceptual loss function as a replacement for the MSE loss function. Compared with MSE, the perceptual loss function focuses more on high-level features such as texture, structure, and content. Specifically, the perceptual loss function leverages a pretrained deep neural network to extract feature representations of images. It calculates the loss based on these feature representations rather than directly comparing pixel-level differences. This approach is more robust to noise and variations in image details, often producing images that are closer to the ground truth data.

In image reconstruction tasks, combining the perceptual loss function with the VGG feature extraction network allows for a more accurate computation of feature differences between the reference and output images. This results in higher-resolution output images. The calculation expression for the perceptual loss function is as follows:

(2) $$loss=\frac{1}{C_{j}H_{j}W_{j}}{\left\|\varphi_{j}\left(\overset{\wedge }{y}\right)-\varphi_{j}\left(y\right)\right\|}_{2}^{2}$$

where $C_{j}H_{j}W_{j}$ represents the size of the feature map at the j−th layer, $\overset{\wedge }{y}$ is the output image, y is the reference image, and φ denotes the loss function. It can be observed that the calculation form of perceptual loss is essentially consistent with MSE, but the calculation domain shifts from the original image space to the feature space of the VGG network. For the feature maps generated at each layer of VGG16, the difference values are computed between the output and reference images. These differences are then used to influence the gradient descent direction of the convolutional neural network via the Adam optimizer. This process iteratively updates the model parameters to achieve an optimal state.

## 3. Data and Experiment

### 3.1. Data Acquisition

Because of the difficulty of obtaining artifact-free ground truth images corresponding to beam hardening artifact images in practical situations, computer simulation was employed to generate the dataset required for network training. The simulation image generation process was as follows:

(1) Simulating CT images of single-material objects: Simulated tomographic images were generated of objects composed of a single material.
(2) Simulating multienergy projection data: Multienergy spectra and material attenuation coefficients were used to simulate the multienergy projection data of the tomographic images.
(3) Reconstruction with filtered back projection: The tomographic images containing beam hardening artifacts using the filtered back projection algorithm.

#### 3.1.1. Artifact-Free Simulated Data

**Figure 4:**
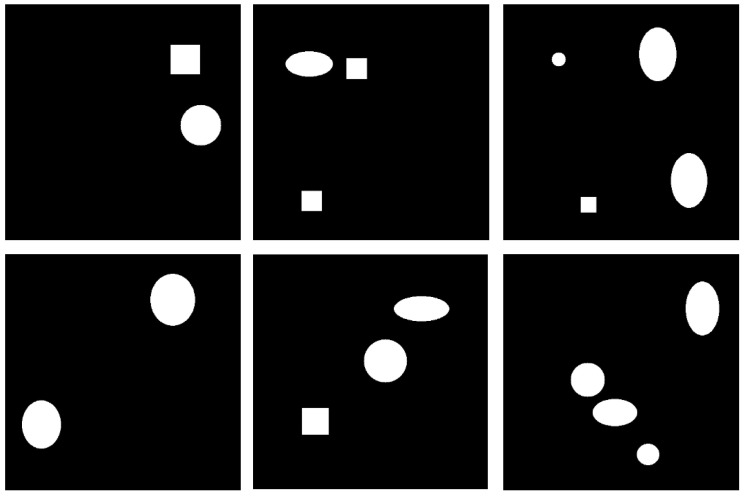
Some simulated ground truth image datasets.

To improve the generalization ability of the network model, a large number of representative data needed to be generated. An artifact-free ground truth image dataset was constructed through random combinations of phantoms with different shapes, including randomly generated circles, rectangles, and ellipses. The image size was set to 512 × 512, with the sizes of the generated patterns being random. The radii of the circles, the lengths and widths of the rectangles, and the major and minor axes of the ellipses were all randomly selected within the range of 20 to 64 pixels. The center points of the patterns were also determined randomly within a square 50 pixels away from the image edges. To avoid overlap between the generated patterns, this experiment set the number of patterns per image to 2, 3, or 4, resulting in 300, 500, and 400 images, respectively, for a total of 1200 images. Of these, 1100 images were used for the training dataset and 100 images were used for the test dataset. Figure 4 shows some of the simulated ground truth images generated.

#### 3.1.2. Generation of Simulated Artifact Data

**Figure 5:**
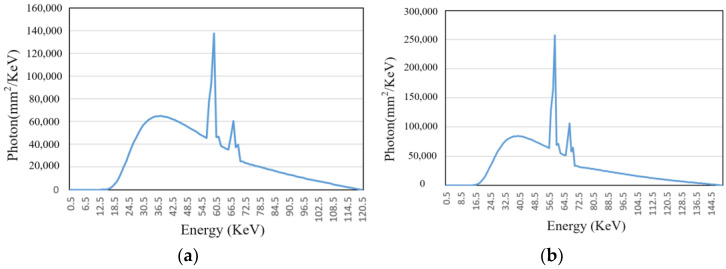
X-ray spectra at two different voltages. (**a**) Energy spectrum curve (120 KV, 2 mA); (**b**) energy spectrum curve (150 KV, 2 mA).

**Figure 6:**
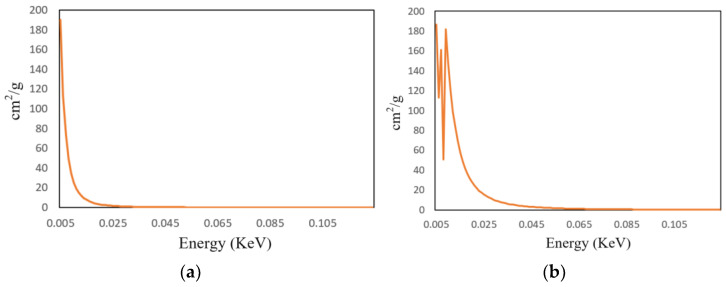
Attenuation coefficient curves of aluminum and copper with energy variation. (**a**) Energy attenuation coefficient of elemental aluminum; (**b**) energy attenuation coefficient of elemental copper.

First, the X-ray energy spectrum at different energies and the attenuation coefficients of the materials used were determined. Based on the empirical energy ranges required for detecting different metal materials, two voltage levels, 120 kV and 150 kV, were selected to simulate the corresponding beam hardening images. The current was set to 2 mA, and the X-ray equipment model used was GE Maxiray 125. The energy spectrum curves can be obtained through Spectrum GUI software 1.0, as shown in Figure 5. Aluminum and copper, two common elements, were chosen as attenuation materials, and their attenuation coefficients can be found on NIST, with the attenuation curves shown in Figure 6.

**Figure 7:**
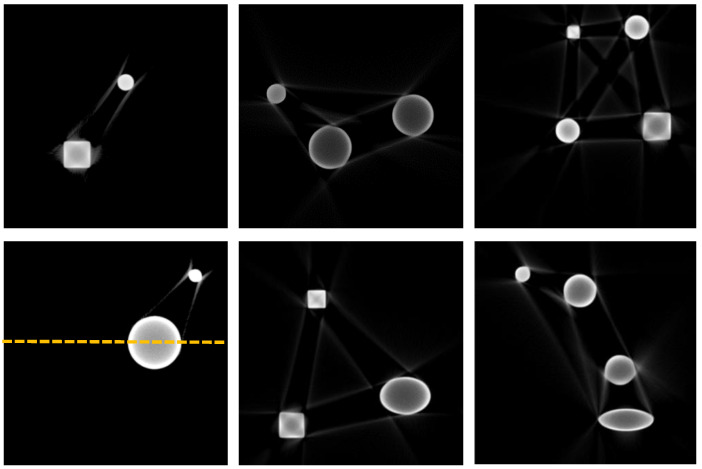
Beam hardening simulation images.

Given the attenuation coefficients of the materials and the energy spectrum curves, the corresponding multienergy projection values for the slice can be calculated. This study adopted the fan-beam FBP reconstruction method, with the image size set to 416 × 416, 640 detector pixels, a scanning angle range of 0° to 360°, and a step size of 0.5° for the scanning angles. The distance between the X-ray source and the detector was 59.5. The simulated tomography images containing beam hardening artifacts, reconstructed using the filtered back projection (FBP) algorithm, were used as the input data for network training. This allowed the network to simulate potential noise and artifact situations encountered in real applications during the training process. The artifact-free images from the above simulation were used as the ground truth training images. Some of the tomography images of the two materials generated under the two voltages are shown in Figure 7.

**Figure 8:**
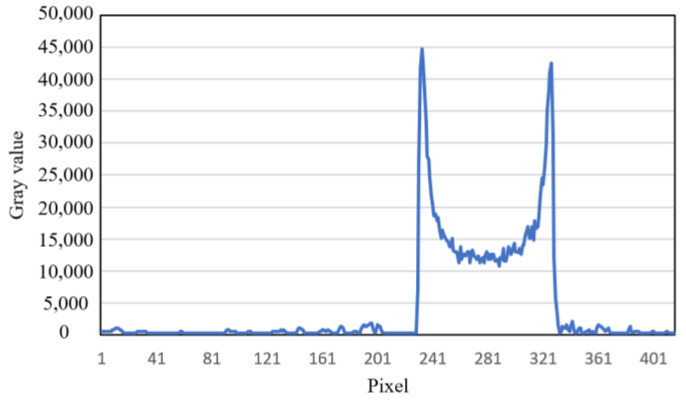
The gray value curve of the row where the yellow line is located in Figure 7.

By observing the simulated image results, it is evident that there was a significant beam hardening phenomenon, with band-like artifacts appearing in the regions between different phantoms. For larger objects, cupping artifacts were observed, with dark centers and bright edges. The gray value curve along the yellow line in Figure 7 is plotted in Figure 8, where the cupping artifact presented by the circular object can be clearly observed. The simulation experiment results closely resembled real beam hardening artifact images.

### 3.2. Network Training

The network training was conducted on the PyTorch 1.5.0 platform, using Python 3.6.5 as the programming language, CUDA version 10.2, and the acceleration library cudnn7.6. The operating system used was Windows 10. The hardware configuration included an Intel Xeon W-3300 CPU, 32 GB of memory, and two NVIDIA Quadro RTX 4000 graphics cards. The network was trained for 200 iterations with a learning rate set to 1 × 10^−4^. The Adam optimization algorithm was used for training, and the weight parameters were initialized according to a normal distribution with a mean of 0 and a standard deviation of 0.1.

## 4. Experimental Results

In this section, we report correction experiments using both simulated beam hardening artifact images and real beam hardening artifact images to validate the method proposed in this paper. We also compare the state-of-the-art CGAN method and the ConvNeXt method with our proposed method. Through subjective visual evaluations and quantitative metric analysis, our method demonstrated optimal image correction results on both simulated and real datasets.

### 4.1. Simulated Experiment Results

**Figure 9:**
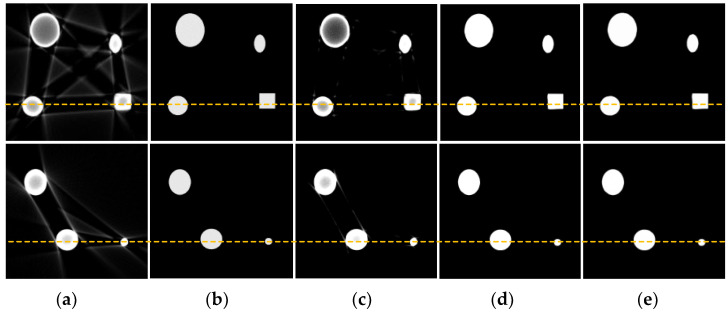
Correction results of simulated images of copper material at 120 kV. (**a**) Artifact image; (**b**) reference image; (**c**) CGAN method; (**d**) ConvNeXt method; (**e**) proposed method.

To validate the effectiveness of the beam hardening artifact correction model, simulated experiments were conducted. The simulated beam hardening artifact image dataset consisted of 1200 images, with 1100 images selected for the training set and the remaining 100 images used as the test set. Figure 9 shows the correction results of the copper material tomography simulated images under 120 kV/2 mA conditions. As can be seen from the figure, the CGAN method removed the bright and dark band artifacts between metals, but the metals themselves still exhibited beam hardening artifacts with dark centers and bright edges. The ConvNeXt method effectively suppressed the artifacts, achieving results close to those of the proposed method in this paper. The algorithm proposed in this paper effectively eliminated the stripe and cupping artifacts caused by the beam hardening effect.

**Figure 10:**
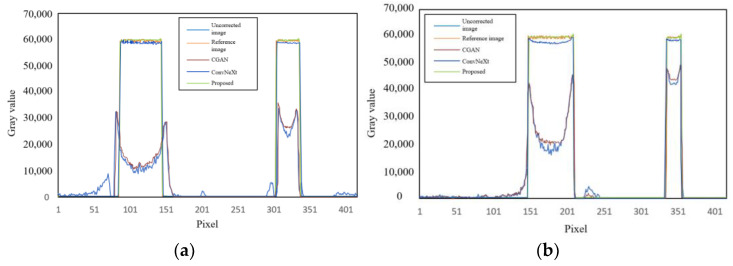
Comparison of gray value curves at the yellow dashed lines in Figure 9. (**a**) Gray value curve of slice 1; (**b**) gray value curve of slice 2.

Additionally, by calculating the gray value curve of any row, the gray value curve results, shown in Figure 10, indicated that the cupping artifacts were well suppressed after correction using this method. Furthermore, the algorithm was also capable of suppressing noise information in the images to some extent, which can be attributed to the downsampling operation in the network. Table 2 shows the quantitative indices for the correction results of the simulated images at 120 kV. The proposed method consistently outperformed CGAN and ConvNeXt across all metrics (RMSE, PSNR, and SSIM), demonstrating its superior ability to correct artifacts, preserve structural details, and enhance image quality. ConvNeXt showed competitive performance but was not as effective as the proposed method, while CGAN lagged significantly behind in all aspects.

**Figure 11:**
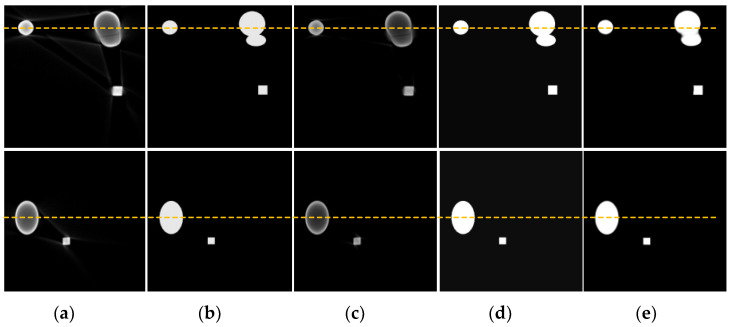
Correction results of simulated images of copper material at 150 kV. (**a**) Artifact image; (**b**) reference image; (**c**) CGAN method; (**d**) ConvNeXt method; (**e**) proposed method.

**Figure 12:**
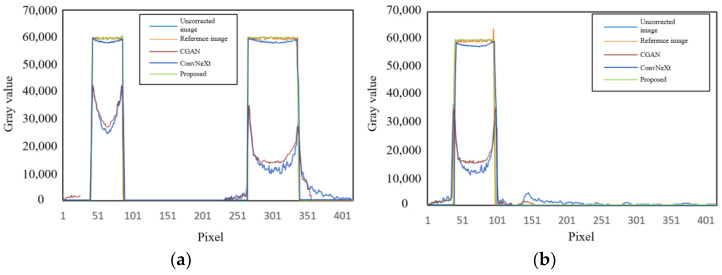
Comparison of gray value curves at the yellow dashed lines in Figure 11. (**a**) Gray value curve of slice 1; (**b**) gray value curve of slice 2.

Figure 11 shows the correction results of beam hardening artifact images under 150 kV/2 mA conditions, indicating that the algorithm still achieved good artifact correction performance under different voltage levels. Although there were differences in the gray-scale range of artifact images generated under different energy conditions, the characteristic patterns of beam hardening artifacts were quite similar. The reason the algorithm achieved good correction results is that the deep learning network can fully learn the obvious characteristic information of beam hardening images and accurately map the artifact images to artifact-free images, benefiting from the network’s training on a large dataset. However, when two objects were close to each other or even overlapped, it could lead to severe structural artifacts, distorting the outer contours of the objects and causing significant edge distortion. As a result, the corrected image could not be fully restored to the artifact-free reference image. Figure 12 shows a comparison of gray value curves at the yellow dashed lines in Figure 11. It can be seen that the proposed method (green line) had the grayscale values closest to the reference image (orange line) at these positions, showing better image restoration effects. The other methods, CGAN (red line) and ConvNeXt (dark blue line), also performed well but were slightly inferior to the proposed method and the reference image. Table 3 shows the quantitative indices for the correction results of the simulated images at 150 kV.

### 4.2. Real Data Experimental Results

#### 4.2.1. Experiment 1: Additive Manufacturing of Titanium Alloy Samples

**Figure 13:**
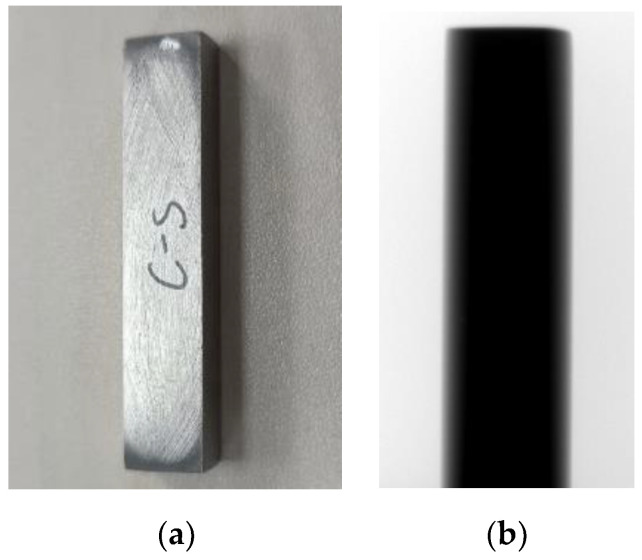
Physical sample and projection of additive titanium alloy. (**a**) Additive titanium alloy sample; (**b**) projection image.

This paper selected two titanium alloy additive manufacturing samples with significantly different sizes for the experiment. First, the method in this paper was validated for beam hardening artifact correction on small-sized samples. Sample 1 had dimensions of 10 mm × 8 mm. An actual image of the sample and its projection image are shown in Figure 13. The tube voltage of the X-ray source was set to 200 kV, and the tube current was set to 2.5 mA. The distance from the X-ray source to the rotation center was set to 292.54 mm, and the distance to the detector was 1622 mm. The specific CT scanning parameters are shown in Table 4.

**Figure 14:**
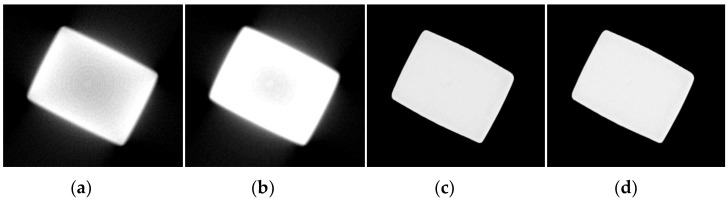
Comparison of the 900th slice of the sample before and after correction. (**a**) Artifact image; (**b**) CGAN method; (**c**) ConvNeXt method; (**d**) proposed method.

After the image reconstruction was completed, a slice from the middle region was randomly selected for testing. The reconstructed image was input into the trained network for model inference, and the result is shown in Figure 14. Table 5 shows the quantitative indices of the correction results for the additive titanium alloy.

From the results of the proposed method, it can be observed that the removal of cupping artifacts in the tomographic image was significant, and the phenomenon of bright edges and dark central regions of the specimen was greatly improved. Since the sample was a single object, there was no occlusion caused by multiple objects, and no stripe-like metallic artifacts appeared in the image. However, there were large areas of scattered artifacts near the edges of the object, which were well corrected, making the object’s edges clearer. The original tomographic image also contained obvious ring artifacts caused by the detector’s inconsistent response to X-rays or pixel channel damage. Although the algorithm in this study did not extract features of the ring artifacts, after correction, the ring artifacts were suppressed, though some severe structured ring artifacts were not completely eliminated.

**Figure 15:**
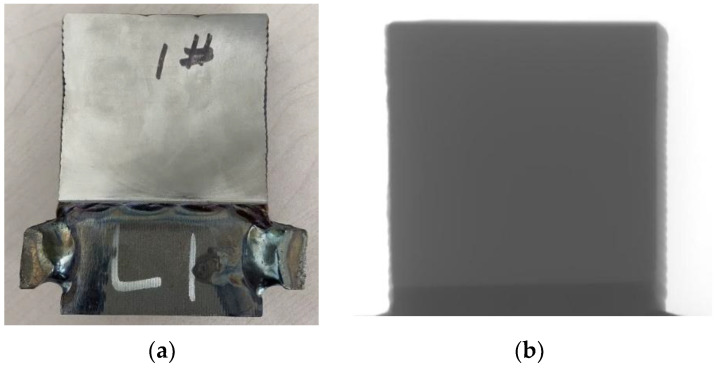
TC17 titanium alloy flat plate sample image. (**a**) Titanium alloy flat plate sample; (**b**) projection image.

**Figure 16:**
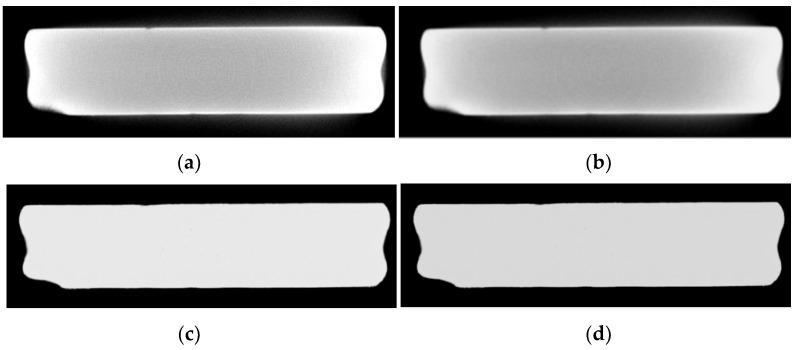
Comparison of the 107th slice image of the flat plate before and after correction. (**a**) Artifact image; (**b**) CGAN method; (**c**) ConvNeXt method; (**d**) proposed method.

Sample 2 was a TC17 titanium alloy flat plate, with dimensions of approximately 100 mm by 25 mm. Actual and projection images of the sample are shown in Figure 15. The upper part of the flat plate was the additive section, the lower part was the substrate, and there were two inserts on the side, with the focus area being the additive section. From the reconstructed image, it is clearly visible that the edges of the object were bright while the internal region was dark, reflecting the typical cupping artifact characteristics. The corrected result using the method in this paper is shown in Figure 16d. Compared with Figure 16a, the contrast of the image corrected with the proposed method was significantly improved, the object’s edges were clearer, and the beam hardening artifact was effectively corrected. Therefore, the experiment results and quantitative index results from Table 6 prove that the algorithm in this paper performed well in correcting beam hardening artifacts for objects of different sizes and under different energy conditions, demonstrating strong network generalization capabilities.

#### 4.2.2. Experiment 2: Blisk Sample

**Figure 17:**
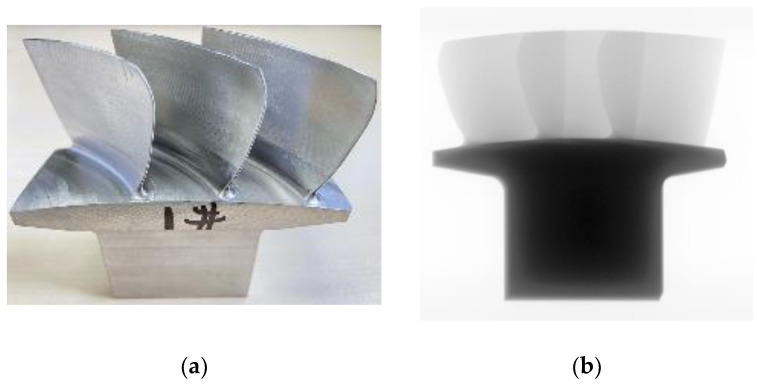
Blisk sample and projection data. (**a**) Blisk sample; (**b**) projection image.

Compared with the traditional blade and disc separate assembly structure, the blisk adopts an integrated design, merging the blades and disc into a single unit. This design not only simplifies the structure by eliminating traditional connecting components such as tenons, mortises, and locking devices but offers performance advantages such as weight reduction, fewer parts, increased efficiency, and improved reliability. In this experiment, a three-component integrated impeller was selected for testing, with the actual sample and its projection image shown in Figure 17.

**Figure 18:**
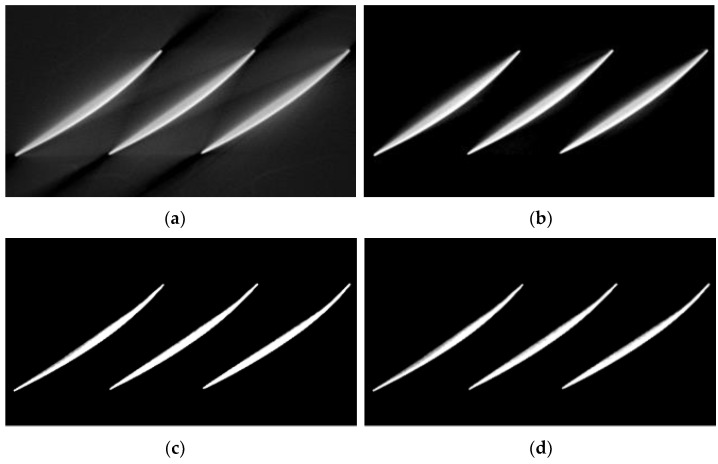
Comparison of the 102nd slice image of the blisk sample before and after correction. (**a**) Artifact image; (**b**) CGAN method; (**c**) ConvNeXt method; (**d**) proposed method.

**Figure 19:**
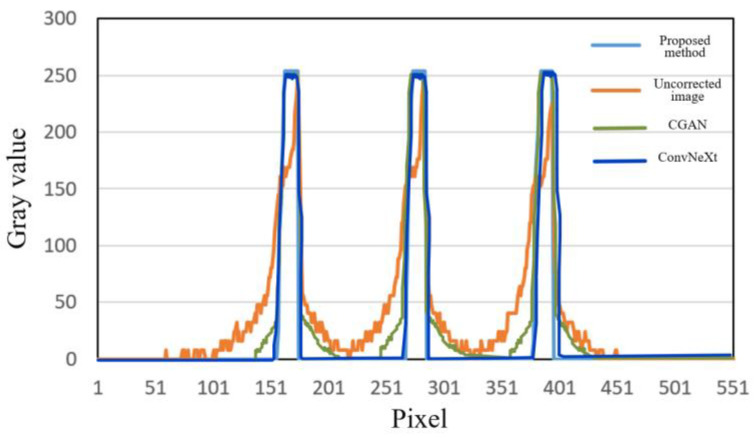
Comparison of gray value curves in the 256th row of the reconstructed images.

The trained network was used to validate the artifact correction effect on the blisk CT image. A comparison between CGAN, ConvNeXt, and the proposed method is shown in Figure 18. In the artifact image, the blades were curved, causing significant beam hardening artifacts in the impeller region, making it difficult to accurately determine the edge of the impeller. Additionally, there was interference from artifacts between adjacent blades, which severely affected the observation and evaluation of internal defects in the blades. The CGAN method removed the bright and dark band artifacts between metals, but the edge structures of the metals remained unclear. After beam hardening artifact correction, most of the artifacts were effectively removed, allowing the true structure of the blades to be accurately represented. In Figure 19, by plotting the gray value curve of row 256 in the image, it can be observed that the artifacts around the impeller were largely eliminated. A comparison of the uncorrected images, the CGAN method, the ConvNeXt method, and the proposed method showed a significant improvement in image quality, with effective suppression of the artifacts and a clearer edge of the impeller, providing a more reliable foundation for observing and evaluating internal defects in the blisk. We also computed the quantitative index results for the blisk, as shown in Table 7.

**Figure 20:**
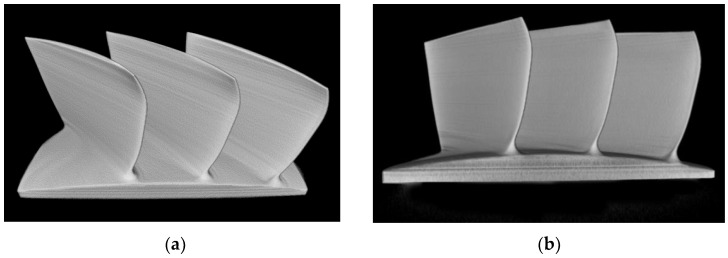
Three-dimensional visualization results of the corrected blisk image. (**a**) Three-dimensional visualization; (**b**) front view.

By performing three-dimensional visualization analysis on the beam hardening corrected tomographic image of the blisk, the integrity of its external structure can be observed and analyzed more intuitively. From Figure 20, it can be seen that the outer contour of the blade was free from beam hardening artifacts and stripe artifacts, with the edges of the blade being clearer and smoother, presenting a more realistic result.

The real data experimental results show that the correction method based on the feature extraction network proposed in this paper could effectively address issues such as cupping artifacts, unclear edge contours, and blurred details caused by beam hardening. It improved the image contrast and enhanced the quality of the CT images.

## 5. Conclusions

This paper focuses primarily on the study of beam hardening artifact correction methods for CT images of aerospace high-density metal materials. It begins with an analysis of the causes of beam hardening and presents the principle of the beam hardening artifact correction algorithm based on feature extraction. A deep convolutional neural network for beam hardening artifact correction was developed, and the structure of the feature extraction network and the perceptual loss function were introduced. A dataset was constructed by simulating beam hardening tomographic images of a large number of single-material samples, and artifact features were automatically extracted from these images. Finally, experimental validation was conducted using both simulated and real beam hardening artifact images. The results demonstrated that the proposed method could effectively address issues such as cupping artifacts, unclear edge contours, and blurred details caused by beam hardening, improving image contrast and enhancing the quality of CT reconstruction images.

## Figures and Tables

**Table 1:** Improved VGG16 framework structure.

VGG-Net					
A	A-LRN	B	C	D	E
11 weight layers	11 weight layers	13 weight layers	16 weight layers	16 weight layers	19 weight layers
Input image (512 pixels × 512 pixels)					
conv-64	conv-64 LRN	conv-64 conv-64	conv-64 conv-64	conv-64 conv-64	conv-64 conv-64
Max pooling					
conv-128	conv-128	conv-128 conv-128	conv-128 conv-128	conv-128 conv-128	conv-128 conv-128
Max pooling					
conv-256conv-256	conv-256 conv-256	conv-256 conv-256	conv-256 conv-256 conv-256	conv-256 conv-256 conv-256	conv-256 conv-256 conv-256 conv-256
Max pooling					
conv-512 conv-512	conv-512 conv-512	conv-512 conv-512	conv-512 conv-512 conv-512	conv-512 conv-512 conv-512	conv-512 conv-512 conv-512 conv-512
Max pooling					
conv-512 conv-512	conv-512 conv-512	conv-512 conv-512	conv-512 conv-512 conv-512	conv-512 conv-512 conv-512	conv-512 conv-512 conv-512 conv-512
Max pooling					

**Table 2:** Quantitative indices for correction results of simulated images at 120 kV.

	Index	CGAN	ConvNeXt	Proposed
Sample 1	RMSE	2.6118	0.8242	0.4156
	PSNR	20.5411	26.7854	28.3647
	SSIM	0.9274	0.9566	0.9638
Sample 2	RMSE	2.8112	1.6514	0.9574
	PSNR	21.9457	24.3789	27.6621
	SSIM	0.9348	0.9470	0.9513

**Table 3:** Quantitative indices for correction results of simulated images at 150 kV.

	Index	CGAN	ConvNeXt	Proposed
Sample 1	RMSE	3.1020	2.5564	1.2128
	PSNR	19.3123	25.7412	26.3147
	SSIM	0.9236	0.9571	0.9634
Sample 2	RMSE	3.2401	2.3697	1.5441
	PSNR	21.0496	25.3412	27.8741
	SSIM	0.9034	0.9367	0.9501

**Table 4:** X-ray CT scanning parameters.

Flat Panel Detector	Pixel Size	Resolution	Ratio	Integration Time	Projection Number
Amorphous silicon	0.139 mm	900 × 900	5.545	1 s	720

**Table 5:** Quantitative indices for correction results of additive titanium alloy.

Index	CGAN	ConvNeXt	Proposed
RMSE	3.1062	1.6472	0.8873
PSNR	25.3601	28.9647	30.1422
SSIM	0.9320	0.9698	0.9731

**Table 6:** Quantitative indices for correction results of flat plate.

Index	CGAN	ConvNeXt	Proposed
RMSE	1.9873	1.0035	0.8423
PSNR	26.3041	27.7415	28.4762
SSIM	0.9598	0.9773	0.9841

**Table 7:** Quantitative indices for correction results of the blisk.

Index	CGAN	ConvNeXt	Proposed
RMSE	3.0193	2.3676	1.3647
PSNR	28.5511	30.6470	33.1478
SSIM	0.9632	0.9796	0.9821

## Data Availability

The data presented in this study are available on request from the corresponding author.
